# Dysfunctional Closed Chest Drainage - Common Causative Factors and Recommendations for Prevention

**DOI:** 10.7759/cureus.2295

**Published:** 2018-03-09

**Authors:** Usman Hashmi, Muhammad Nadeem, Abdul Aleem, Fuad Ul Hasan H Khan, Rabeea Gull, Kaleen Ullah, Iftikhar H Khan

**Affiliations:** 1 Thoracic Surgery, Nishtar Medical University/hospital Multan, Pakistan.; 2 Surgery, Buffalo General Hospital

**Keywords:** dysfunctional chest drainage unit, faulty chest tubes, non-functioning tube thoracostomy, failure of chest drainage system

## Abstract

Introduction

Dysfunctional closed chest drainage unit (CDU) dysfunction is a common but serious clinical problem associated with tube thoracostomy and results in a significant rise in morbidity, prolonged hospital stays, and increased economic burden. This observational study examines the proximate factors of closed CDU dysfunction in addition to their relative frequency. Based on our findings, we suggest logical recommendations for preventing the factors that contribute to closed chest drainage unit dysfunction.

Method

The study target population consists of all those individuals who had experienced tube thoracostomy for any pathology related to the chest cavity treated in the Department of Thoracic Surgery, Nishter Medical University, Multan, Pakistan, from February 2015 to January 2017. The study population was not restricted by age or gender. Of the 727 examined cases, only those patients who had experienced tube thoracostomy and had significant failure in draining the pleural collection were included in the study. Detailed histories were collected, and thorough physical examinations were carried out for each participant. Chest x-rays and, if needed, computed tomography (CT) scans were obtained to properly examine the placement of the chest tubes and detect the causative factor of the closed CDU dysfunction.

Results

A total of 139 cases were included in the study. The most common cause of closed CDU dysfunction was the use of the wrong CDU connection (n = 24, 17.3%). Other common problems included inadequate prime fluid use, loose connections, kinked tubes, and overly full bottles.

Conclusion

Closed CDU dysfunction may be prevented by adopting and following proper protocols for tube thoracostomy.

## Introduction

Tube thoracostomy (TT) is a common surgical procedure that can be performed at the bedside, as well as in an operating room, and is indicated in life-threatening emergencies and for postoperative chest drainage in elective surgery [1]. TT is a life-saving technique and, in many situations, no alternative treatment exists [2]. However, despite the potential benefits, the TT procedure is associated with life-threatening complications, such as laceration of thoracic and abdominal viscera, injury to the great vessels, and bronchopleural fistula [3-4]. The cumulative rate of these complications is approximately 3% to 22% [1, 3, 5]. Sometimes what appears to be a perfectly placed TT may be nonfunctioning due to disturbed negative pressure within the chest drainage unit (CDU). The basic design principle of these CDUs is to generate and maintain the negative pressure by preventing air entry within the pleural space for constant and effective drainage of any pleural collection [6]. Therefore, any factor causing an impairment of the negative pressure in the CDU will result in its dysfunction. CDU dysfunction is a commonly encountered clinical problem. This article focuses on the factors causing CDU dysfunction and provides recommendations to prevent said factors.

## Materials and methods

This observational study was conducted in the Department of Thoracic Surgery at Nishter Medical University Multan, Pakistan from February 2015 to January 2017. The target population was composed of all those individuals who had experienced TT for any pathology related to the chest cavity. A total of 727 patients were examined in which TT was performed for postoperative chest drainage, pleural effusion, flail chest, tension pneumothorax, spontaneous pneumothorax, empyema thoracic, hydropneumothorax, haemothorax, and chylothorax. Moreover, the study population was not restricted by age or gender. Only those patients who had experienced TT with significant failure in draining the pleural collection were included in the study. The other inclusion criteria were a) persistent air leak and failure of the lung to re-expand following the insertion of one or more TTs; b) failure in the drainage of intrapleural fluid or blood in patients with empyema or hemothorax despite placement of one or two TTs; and c) TT failure within seven days.

Patients with loculated empyema, bronchopleural fistula, and dislodged TT were not included. A detailed history and thorough physical examination that included an assessment of the CDU from the insertion site to an underwater seal bottle was carried out for each participant. Preoperatively, required investigations related to the specific lung pathologies were carried out, such as chest x-ray, ultrasound of the chest, computed tomography (CT) scan of the chest, pleural fluid analysis, tuberculosis (TB) skin test, sputum examination for TB, TB interferon-gamma release assays, and fine needle aspiration cytology or Tru-Cut biopsy of the mass to establish the diagnosis. Postoperatively, a chest x-ray and, where necessary, a chest CT scan was used to evaluate the placement of the TT and assess lung pathologies, such as loculated empyema and bullous diseases of the lungs. We assessed these patients to explore factors causing dysfunctional closed CDU and documented all possible complications and mistakes related to chest tube insertion and care of its system. Patients’ demographic data were recorded on a Performa. All statistical analysis was done in IBM SPSS Statistics for Windows, Version 19.0 (Released 2010, IBM Corp., Armonk, NY). The mean and the standard deviation were computed for numerical variables like age. Frequencies and percentages were computed for categorical variables, such as gender and causative factors of CDU dysfunction. 

## Results

In 139 cases, 36 (25.9%) were women while 103 (74.1%) were men. The mean age of our study cases was 37 ± 14 years (range: 12 to 65 years). Table 1 contains demographic information for the study participants.

**Table 1:** Demographic Characteristics in All Patients

Total (n)	139
Mean Age (Years)	37 ± 14
Gender	Male: 103
	Female: 36

A wrong connection of CDU was the most common cause of dysfunctional closed CDU (n = 24; 17.3%). Other common problems involved underwater seals, inadequate prime fluid, loose connections, kinked tubes, and overfull bottles. Table 2 lists the causes recorded for the study along with their relative frequency.

**Table 2:** Relative Frequency of Different Causes in Patients

Causes	Frequency	Percentage
Wrong Connections	24	17.3%
Odd Underwater Seal	19	13.7%
Inadequate Prime Fluid	15	10.8%
Loose Connections	15	10.8%
Overfull Bottles	12	8.6%
Kinked Tubes	10	7.2%
Clamping	7	5.0%
Holes in Tubes	7	5.0%
Sealed off Vent	7	5.0%
Faulty Suction	5	3.6%
Eyelet out of Pleural Space	5	3.6%
Bottle Above Level of Chest	5	3.6%
Absent Drainage Bottle	2	1.4%
Subcutaneous Chest Tube	2	1.4%
Odd Chest Tube	2	1.4%
Stitch Cutting Through the Tube	2	1.4%
Total	139	100%

## Discussion

Dysfunctional closed CDU is a common condition which can lead to grave consequences if not managed promptly. In the literature, various causes of a CDU system failure are described as shown in Table 3.

**Table 3:** Frequency of Causative Factors of Dysfunctional CDU CDU: chest drainage unit; TT: tube thoracostomy

Causative Factor	Relative Frequency
Ectopic TT	23% - 53% [7]
Clamping	9.1% [8]
Faulty suction system	6.8% [7]
Loose connections	4.4% [7]
Tube blockage	0.6% - 3.5% [9-10]
Sealed-off vent	3.2% [7]
Intrathoracic tube kinking	2.7% - 3.9% [11]
Subcutaneous emphysema	1% [9-10]
Improper filling of the underwater seal bottles	0.3% [7]
Subcutaneous placement of tube	1% - 1.8% [12]

We found faulty connections of CDU tubing was the most frequent cause of CDU dysfunction. In other studies, ectopic TT and clamping of TT were the most common pitfalls in TT placement [7-8]. Loose CDU connections, improper filling of underwater seal bottle, and sealed-off vents were more frequent in our study than what was reported by Al-Tarshihi et al. [7].

Faulty connections and improper underwater seal bottle can defeat the purpose of TT and result in a pneumothorax. When the tube from the patient’s chest is connected to the vent and the bottle system is sealed off, the patient may have tension pneumothorax because the air coming from patient’s chest has nowhere to vent. If the bottle system is not sealed off, the patient is practically in open pneumothorax because the end of the tube coming from the patient’s chest is in free communication with the environment [13]. Therefore, the tubing connections should be vigilantly performed, and a senior team member should cross-check the integrity and accuracy of the connections. Multiple connections should be avoided, and the ends of the connection pieces should be serrated/stepped. No connection of the CDU should ever be covered with an adhesive tape [14].

After finishing the TT placement, the proper functioning of CDU system must be confirmed. It can be done by observing the “swinging” of the water column with respiration. Moreover, at least 4 cm of tube length coming from the patient’s chest should be immersed underwater to protect against the end of the tube coming out of the water in case of bottle tilt [14]. Appropriate levels (i.e., 4 cm to 5 cm) of prime fluid should be maintained in the bottle of the CDU. It should not be allowed to build up beyond 15 cm. If it is higher than 15 cm, then a status of relative tension builds up in the system, and at levels higher than 25 cm, features of tension pneumothorax can appear if there is an air leak from the lung [15]. We observed that the bottle of the CDU was present at or even above the level of patient’s chest on occasion. This mistake was commonly observed while transferring the patient from one department to another or during mobilization of the patient. The fluid in the underwater seal is not sterile, and its retrograde flow into the pleural space can result in empyema thoracis. If chemicals are present in the solution, severe chemical pleuritis will further complicate the condition. Moreover, if a significant amount of bottle fluid siphons into the chest, there is a possibility that not enough fluid is left in the bottle to maintain the underwater seal, and the end of the chest tube, which was immersed underwater, comes to lie above the water level resulting in a state of open pneumothorax. To prevent this serious complication, the patient and the family should be properly educated regarding the handling of the CDU. They should be directed to keep the CDUs below the level of the chest. The flutter valve systems may be used in patients who are stable with mild air and fluid leak from the TT [16]. Only sterile 0.9% saline solution should be used as the prime fluid in the underwater seal bottles. Moreover, antiseptic solutions should never be added to the prime fluid because these are strong irritants, and their retrograde flow can result in chemical pleuritis [17]. Usually, the use of suction is advocated in cases of persistent air leak and non-resolving pneumothorax [12]. However, we found that a faulty suction system or turning the suction off without disconnecting it from the drainage bottle was inhibiting the lung re-expansion and drainage of the pleural space collection. This can be prevented by using a high-volume, low-pressure suction system [18]. It is important to note the suction system is functioning properly. When suction is no longer needed, it should be detached from the CDU bottle.

Similarly, a kinked or angulated tube impairs the efficacy of TT and can cause poor drainage, discomfort, and trauma on removal, leading to the collection of undrained pleural fluids [19]. A study conducted by Adame et al. reported kinked intrathoracic tubing in 2.7% to 3.9% of cases in their study [11], which was much lower than the incidence of kinking we found. The recent medical literature recommends that Mac technique testing should be implemented while performing a TT placement to determine the presence of kinking. It consists of grasping the external portion of the TT, turning it clockwise at 180 degrees, and then releasing the tube. If the tube restitutes back to its original position, the test is considered positive, and the tube is considered to be kinked. If the tube does not spin back and stays in its position upon release, the test is considered negative [11, 20].

Another course of action is to use a blunt, malleable sterile stylet inside the tube at the time of insertion. It is inserted into the chest tube so that it does not protrude beyond the tube and is 1 cm short of the tip to avoid visceral injury by the stylet [21]. The incidence rate of factors responsible for the CDU dysfunction was very high in our study compared to the incidence rate reported in the literature [22-23]. However, subcutaneous placement of the TT was present in only 1.4% cases of our study, which is comparable to a European study that reported an incidence rate of 1 - 1.8% [12]. Problems related to the position of the chest drain account for a heavy proportion of CDU dysfunction occurrence [7]. When the sentinel hole or the eyelet of TT is present outside the pleural cavity, it creates a communication between the chest cavity and subcutaneous tissue and causes surgical emphysema. Furthermore, the presence of a sentinel hole or eyelet outside the skin wound exposes the patient in a state of open pneumothorax [12]. The incidence rate of this common but serious complication can be reduced by adopting the proper technique of TT placement. To prevent subcutaneous placement, an adequately sized incision will allow full dissection through subcutaneous tissue and intercostal muscles [24]. Prior to entering the pleural space, a sterile gloved finger should be passed through the dissected tract to confirm that pleural space has been entered. Moreover, the approximate length of the TT to be inserted into the pleural cavity should be measured before insertion [24]. There should be a distance of at least 5 cm between the last eyelet of the chest drain and the entry point of the chest drain into the chest wall [25]. In general, no extra holes should be created in the chest drain. If it is necessary to create extra holes, then the most distal holes should be created across the radiopaque line. The fixation stitch on the tube should be tied with the patient’s arm in adduction [26]. If a chest tube is fixated with the patient’s arm in full abduction (especially in obese patients) and the patient then adducts his arm after fixation, the fixation stitch moves caudally with the folds of skin, and it pulls the tube down, which may lead to the last eyelet coming out of the pleural space. The fixation stitch should never be passed through the chest tube because it creates a tiny communication through the hole between the negative intrapleural and the atmospheric pressure.

In low-quality CDUs, the tubes used in the system were neither of medical grade nor of good quality material. These tubes get cracked when repeatedly clamped or milked with a roller. Once the tube gets cracked, the negative pressure inside the CDU is lost, and the patient goes into open pneumothorax as the CDU is practically in communication with the environment through the crack in the tube. Therefore, unnecessarily repeated clamping of the tubing should be avoided [27]. If a clamp is applied at all, then on the removal of the clamp, careful examination of the tube should be done to rule out any structural damage to the tube at the site of clamping. The use of rubber tipped or padded clamps is less likely to cause structural damage to the tube. 

We observed empty bottles of intravenous (IV) fluids, ordinary drainage bags (with no Heimlich valve), and even balloons and plastic shopping bags used as drainage bottles. In two cases, no drainage bottle was present, and the chest tube was open to the air. These conditions lead to system failure. Therefore, only a standard, properly designed CDU system should be used. Odd and inappropriate chest tubes (e.g., Foley catheters, nasogastric tubing, and IV infusion sets) should never be used in place of standard chest drains. The manufacturer’s instructions should be closely followed in setting up the CDU system.

Our observations revealed an alarming situation. Even in a tertiary level hospital, the knowledge and training of medical staff are not adequate, and numerous serious problems result due to these shortcomings in TT placement and management of CDUs. Nurses and the residents should be properly trained regarding management of chest drains to avoid lethal complications.

## Conclusions

Dysfunctional closed CDU is a common but serious clinical problem that usually results from wrong or inadequate connections to the CDU system. Inadequate levels of prime fluid, loose connections, kinked tubes, and overfull bottles are other major causative factors of CDU dysfunction. These mistakes in the handling of the TT and CDU system are due to inadequate knowledge and poor levels of experience for nurses and residents. Therefore, training courses for both the residents and the nurses should be mandatory in any hospital dealing with thoracic patients. Only standard, specifically designed chest tubes and CDUs should be used. Furthermore, the residents and paramedical staff should be educated and trained in how to follow the proper protocol of TT and management of CDU systems to prevent this life-threatening and disastrous condition.
